# s-SHIP expression identifies a subset of murine basal prostate cells as neonatal stem cells

**DOI:** 10.18632/oncotarget.8709

**Published:** 2016-04-12

**Authors:** Guillaume Brocqueville, Renee S. Chmelar, Hélène Bauderlique-Le Roy, Emeric Deruy, Lu Tian, Robert L. Vessella, Norman M. Greenberg, Larry R. Rohrschneider, Roland P. Bourette

**Affiliations:** ^1^ University of Lille, CNRS, Institut Pasteur de Lille, UMR 8161-M3T-Mechanisms of Tumorigenesis and Targeted Therapies, SIRIC ONCOLille, F-59000 Lille, France; ^2^ Division of Basic Sciences, Fred Hutchinson Cancer Research Center, Seattle, WA 98109, USA; ^3^ BioImaging Center Lille, Institut Pasteur de Lille, University of Lille, F-59000 Lille, France; ^4^ Department of Urology, University of Washington, Seattle, WA 98195, USA; ^5^ Present address: NMG Scientific Consulting, North Potomac, MD 20878, USA

**Keywords:** stem cells, prostate, s-SHIP, epithelial cells, transgenic mouse

## Abstract

Isolation of prostate stem cells (PSCs) is crucial for understanding their biology during normal development and tumorigenesis. In this aim, we used a transgenic mouse model expressing GFP from the stem cell-specific s-SHIP promoter to mark putative stem cells during postnatal prostate development. Here we show that cells identified by GFP expression are present transiently during early prostate development and localize to the basal cell layer of the epithelium. These prostate GFP^+^ cells are a subpopulation of the Lin^−^ CD24^+^ Sca-1^+^ CD49f^+^ cells and are capable of self-renewal together with enhanced growth potential in sphere-forming assay *in vitro*, a phenotype consistent with that of a PSC population. Transplantation assays of prostate GFP^+^ cells demonstrate reconstitution of prostate ducts containing both basal and luminal cells in renal grafts. Altogether, these results demonstrate that s-SHIP promoter expression is a new marker for neonatal basal prostate cells exhibiting stem cell properties that enables PSCs *in situ* identification and isolation via a single consistent parameter. Transcriptional profiling of these GFP^+^ neonatal stem cells showed an increased expression of several components of the Wnt signaling pathway. It also identified stem cell regulators with potential applications for further analyses of normal and cancer stem cells.

## INTRODUCTION

Most tissues contain a small dedicated stem cell population, which is essential for maintaining tissue homeostasis and for tissue repair after injury [1, 2]. Adult tissue stem cells also may contribute to cancer development as being the cells of origin in cancer or tumor-initiating cells [3]. Therefore, these adult stem cells provide an enormous advantage to survival of the adult organism through tissue maintenance, regeneration and repair; but at the same time, they may represent a risk to the tissue or organism due to potential cancer development [4]. Thus, identification and characterization of populations of normal adult epithelial stem cells are major goals and represent the first steps towards understanding normal versus abnormal tissue physiology, ultimately leading to new therapeutic approaches for diseased or injured tissues.

The prostate gland is a complex structure made of branched epithelial ducts within a stromal matrix, which offer an excellent system for studying the function and regulation of epithelial stem cells during early development, sexual maturity and tumorigenesis. The mouse prostate epithelium is comprised of two major cell types: a layer of tall columnar secretory cells surrounding the lumen and a discontinuous layer of flattened basal cells in close contact with the basement membrane separating epithelial cells from its stroma. These two cell types originate from the initial solid epithelial bud that emerges from the urogenital sinus epithelium and elongates into the surrounding mesenchyme through intensive proliferation at their ductal tips [5]. Another rare and not well–understood cell type is the neuroendocrine cell, dispersed within the basal layer [5].

Stem cells responsible for prostate tissue regeneration were first suggested upon androgen deprivation (castration) in animal studies. Castration leads to rapid involution of the prostate gland, but once androgen is provided back, the prostate completely regenerates, and this cycle of involution–regeneration can be repeated more than 30 times [6]. Further studies demonstrated that prostate stem cells (PSCs) resided within the proximal region nearest the urethra [7–9]. Lineage tracing experiments generated recent insights in epithelial cell hierarchy in the mouse prostate, using different cytokeratin promoters to mark either basal or luminal cells. During the first stage of prostate development, from birth to the beginning of puberty, multipotent stem cells are located in the basal cell lineage with the potential of differentiation into basal, luminal and neuroendocrine cells [10, 11]. In adult prostate, bipotent stem cells exist but are scarce both in basal and luminal lineages, and adult epithelia are mainly maintained by respective unipotent stem/progenitor cells within the basal and luminal cell lineages [12–14].

The ability to isolate these PSCs is crucial for the in-depth study of their biology and involvement in development and cancer. Using combinations of cell-surface markers, fractionated mouse PSCs have been identified as Lin^−^Sca-1^+^ CD133^+^ CD44^+^ CD117^+^ proximal cells [15], Lin^−^Sca1^+^ CD49f^+^ [8] and Lin^−^Sca1^+^ CD49f^high^ Trop2^high^ basal cells [16–18]. Similar approaches have been used with human cells using combinations of different makers such as α2β1 integrin [19], CD133 [20], CD44 [21], CD166 [22] and Trop2 [16, 23, 24].

Due to the general lack of unique cell-surface markers, in particular markers that allow *in situ* stem cell identification, the development of new markers to prospectively identify putative stem cells is of the utmost importance for exploring the dynamics, function, and regulation of stem cells. Stem cell-specific expression of s-SHIP was initially identified in embryonic and hematopoietic stem cells [25]. A transgenic mouse model (Tg 11.5kb–GFP) was generated using the 11.5kb s-SHIP promoter and we found that the s-SHIP promoter specifically expressed enhanced green fluorescent protein (GFP) in several potential stem cell populations in embryonic development, including the skin epidermis, hair follicles, mammary gland, and prostate [26]. In the postnatal mammary gland, we showed that GFP labels puberty cap cells and pregnancy basal alveolar bud cells, and demonstrated that they are activated mammary stem cells [27]. In the 11.5kb-GFP transgenic mouse embryo, s-SHIP/GFP is expressed in prostate bud morphogenesis at E18.5 days [26]. In the present study, we investigated s-SHIP/GFP expression in neonatal and adult prostate of Tg 11.5kb-GFP mice and we showed that s-SHIP/GFP-expressing prostatic epithelial cells represent a subset of neonatal basal epithelial cell population with stem cell properties. These cells localize to the basal region during ductal canalization, exhibiting a Lin^−^ CD24^+^ Sca-1^+^ CD49f^+^ basal phenotype, are enriched for prostate sphere-forming activity *in vitro*, and can regenerate prostatic tubules *in vivo*. These results demonstrate that s-SHIP promoter expression offers a valuable marker of stem cell populations for both mammary and prostate tissues.

## RESULTS

### s-SHIP promoter is expressed transiently during early mouse prostate development

**Figure 1:**
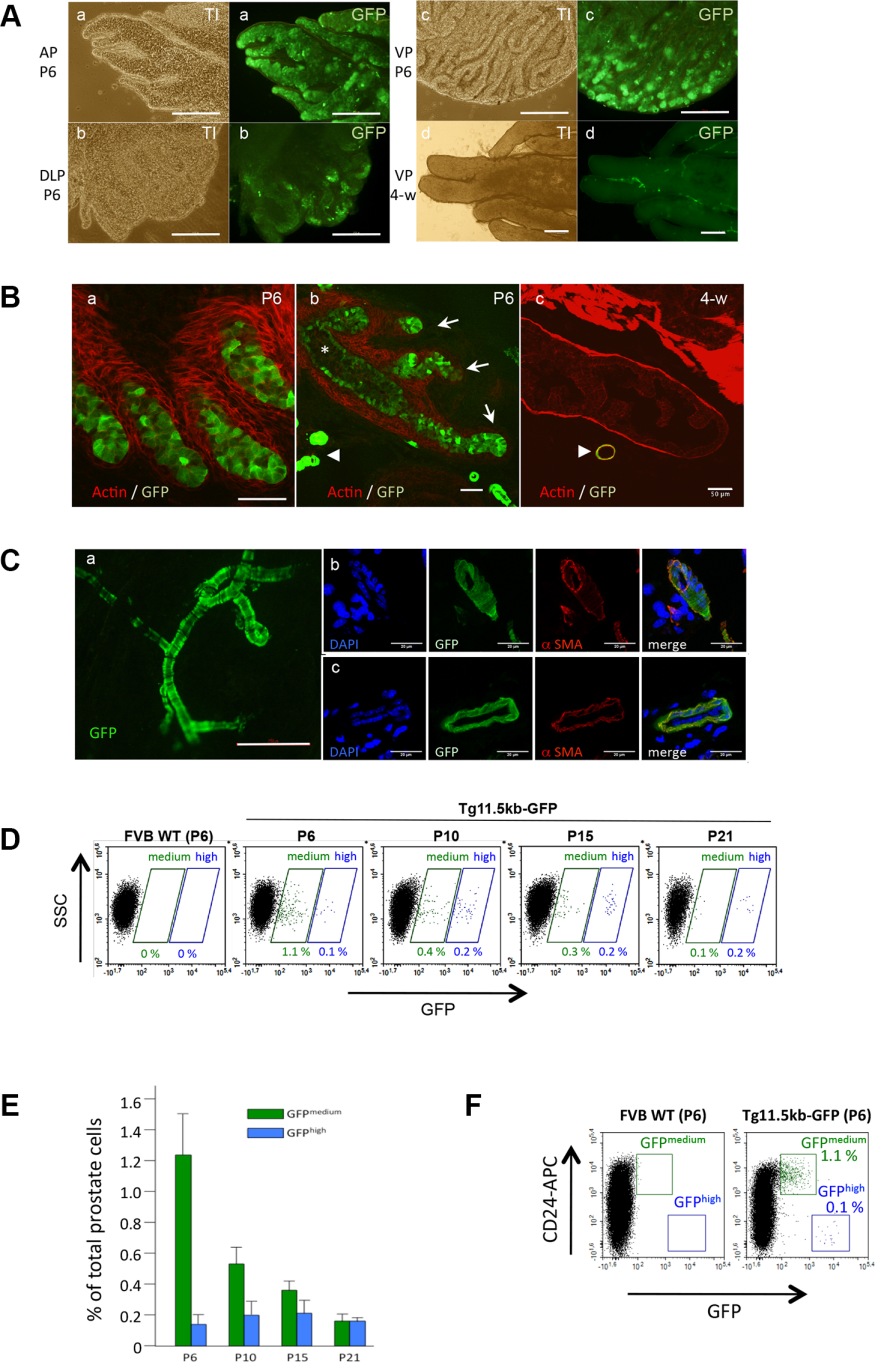
s-SHIP/GFP is transiently expressed in epithelial ducts during postnatal prostate development (**A**) Whole mount of anterior (AP), dorsolateral (DLP), and ventral (VP) prostate lobes of Tg 11.5kb-GFP mice, 6 days (P6) (a-c) or 4 weeks (4-w) (d) after birth were imaged under a fluorescence microscope. Representative picture (*n* = 3) of 4-w ventral lobe (d) is characteristic of all 4-w lobes. (**B**) Representative photographs (*n* > 10) of frozen sections of P6 (a,b) or 4-w (c) prostate tissues from Tg 11.5kb-GFP mice stained with phalloidin-Alexa594 for polymerized actin (red) to show the glandular architecture. (**C**) Representative photograph (*n* > 10) showing the typical morphology of blood vessels in Tg 11.5kb-GFP mice with GFP^+^-vascular smooth muscle cells (a). These vessel-associated GFP^+^ cells stained for alpha smooth muscle actin (b, c) (**D**) Representative flow cytometry analysis (*n* = 3) of GFP expressed by dissociated prostate cells isolated from P6 to P21 Tg 11.5kb-GFP mice. (**E**) Bar graph shows the frequency of GFP^medium^ (green bars) and GFP^high^ (blue bars) cells in total dissociated prostate cells. Data represent the mean ± s.d., *n* = 3. (**F**) Representative flow cytometry (*n* > 5) analysis of GFP expressed by dissociated prostate cells isolated from P6 wild-type FVB (left panel) or P6 Tg 11.5kb-GFP (right panel) mice and labelled with anti-CD24 (APC) pan-epithelial marker. Transillumination (TI), side-scatter (SSC), allophycocyanin (APC), alpha smooth muscle actin (αSMA). Scale bars : 250 μm (A, Ca), 50 μm (B). 20 μm (Cb,c).

In postnatal 6-day-old (P6) prostate, GFP expression was detected in all prostatic lobes with a majority of GFP^+^ cells located in the distal tips of the solid cords (Figure 1Aa–c), which elongate into surrounding mesenchyme as a result of intense proliferative activity [28]. GFP expression was detected in groups of cells located in the terminal ductal tips and ductal branch points (Figure 1Ba–b, arrows) but intensity decreased in differentiating cells as the solid epithelial cords canalize (Figure 1Bb, asterisk). When the prostate gland matured, s-SHIP promoter expression progressively turned off in most of the growing buds, until virtually no GFP-expressing cells was observed in 4 week-old prostate (Figure 1Ad, Bc) and at later stages of development (not shown). The only GFP-expressing cells observed in prostate tissue throughout the prostate development and in the adult were those associated to blood vessels surrounding the epithelium. In these cells, GFP expression was more intense than in epithelial cells (Figure 1Bb–c, arrow-heads, 1Ca). These vessel-associated GFP-expressing cells expressed alpha smooth muscle actin (αSMA) (Figure 1Cb, c) and have been previously characterized in the Tg11.5kb-GFP mice as a subpopulation of vascular smooth muscle cells (vSMCs) [26, 27].

To quantify the percentage of GFP^+^ cells during early mouse development, prostate tissue was harvested at different ages and digested by collagenase to prepare dissociated single-cell suspensions. GFP expression was analyzed and quantified by flow cytometry (Figure 1D, 1E). As compared to P6 wild-type FVB mice, two distinct GFP^+^ cell populations were present in Tg11.5kb-GFP prostate: a cell population with a medium GFP expression level (GFP^med^) that decreased rapidly after birth, from 1.23% ± 0.31 of the total cells at P6 to 0.16% ± 0.04 of the total cells at P21, and a distinct group of cells exhibiting higher GFP expression (GFP^high^) that was present at all stages of prostate development, with similar cell numbers from P6 to P21, suggesting that these cells corresponded to the GFP^+^-vSMCs (Figure 1D, 1E). The non-epithelial phenotype of these GFP^high^ cells was confirmed using the pan-epithelial cell marker CD24 (also called heat-stable antigen) [8] that discriminated CD24^+^ GFP^med^ epithelial cells from CD24^−^ GFP^high^ non-epithelial cells (Figure 1F). Thus, s-SHIP promoter was expressed in a rare cell population, mainly during the first two weeks after birth, which correspond to intense morphogenetic activity. We therefore focused our study on these postnatal P6 epithelial GFP^med^ cells.

### GFP^+^ cells localize to the basal region of the prostate epithelium

**Figure 2:**
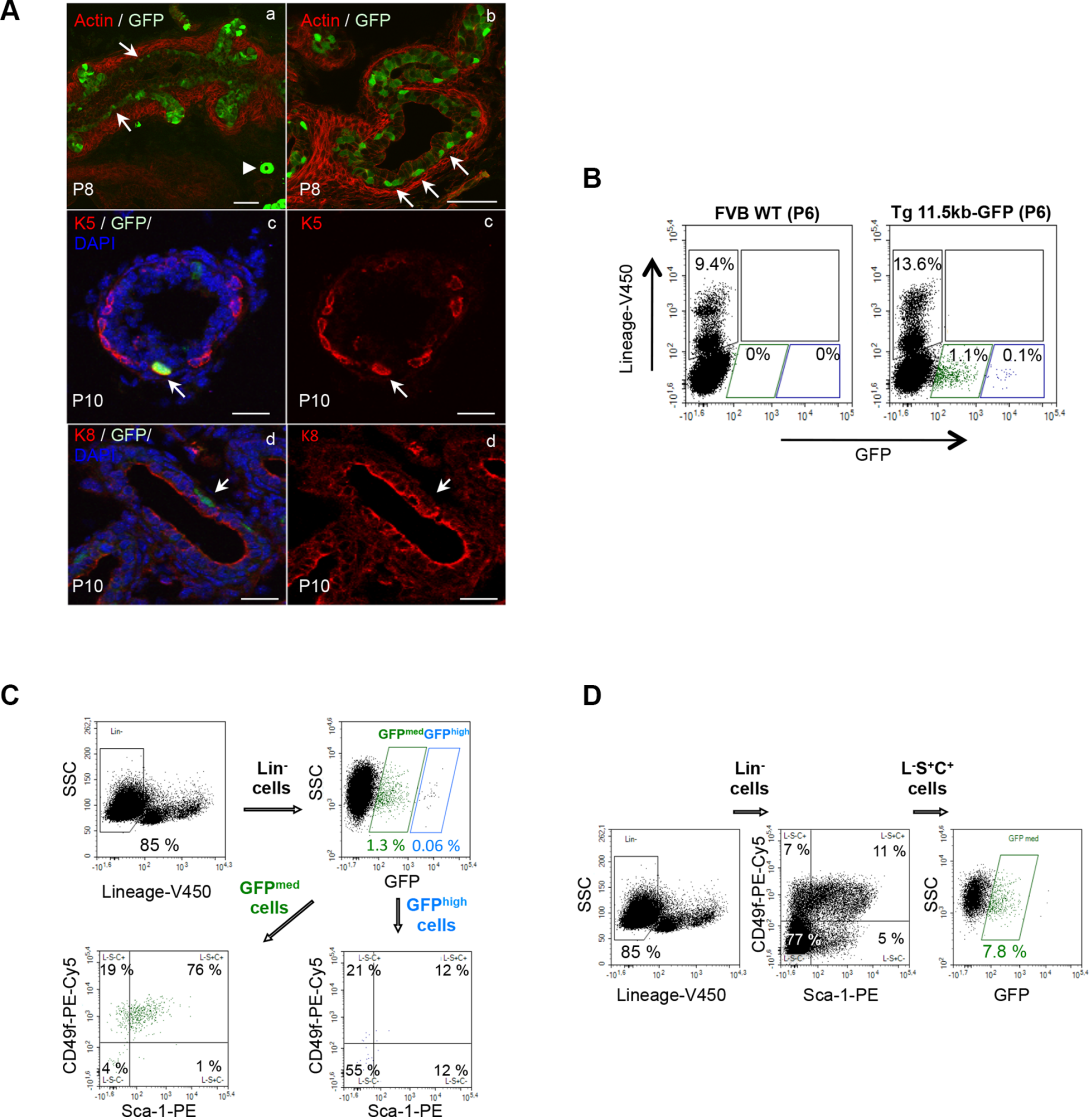
s-SHIP/GFP-expressing cells are a subset of neonatal basal prostate cells (**A**) s-SHIP/GFP-expressing cells localized into the basal region of the prostate epithelium in the differentiating ducts of Tg11.5kb-GFP mice; representative photographs (*n* > 10) of frozen sections of P8 prostate tissues from Tg 11.5kb-GFP mice stained with phalloidin-Alexa594 for polymerized actin (red) to show the glandular architecture (a,b); arrows indicate GFP^+^ cells located in close contact with the basement membrane and the arrowhead indicates a blood vessel. Representative photographs (*n* > 5) of immunofluorescent staining (red) of P10 frozen prostate sections for basal cell marker cytokeratin 5 (K5) (c) and luminal cell marker cytokeratin 8 (K8) (d); arrows mark K5^+^ GFP^+^ (c) or K8^−^ GFP^+^ (d) cells. Sections were counterstained with DAPI nuclear stain (blue) (c,d). (**B**) Both GFP-expressing cell populations were negative for lineage cell surface markers; representative flow cytometry analysis (*n* = 3) of dissociated prostate cells isolated from P6 wild-type FVB (left panel) and P6 Tg 11.5kb-GFP (right panel) mice and stained with V450-conjugated antibodies against non-prostate cell lineage markers (CD31, CD45, and TER119). (**C**, **D**) s-SHIP/GFP^med^ expression identifies a subpopulation of L-S+C+ prostate basal cells. Representative flow cytometry analysis (*n* > 10) of dissociated prostate cells isolated from P6 Tg 11.5kb-GFP mice and stained with V450-conjugated antibodies against non-prostate cell lineage markers (Lin), with PE-conjugated antibodies anti-Sca-1 and PE-Cy5-conjugated antibodies anti-CD49f. FACS plots show gates drawn for sequential analysis of (C) Sca-1 and CD49f cell surface marker expression on Lin^−^ GFP^high^ and Lin^−^ GFP^med^ cell subpopulations, and (D) GFP expression on Lin^−^ Sca-1^+^ CD49f^+^ (L-S+C+) cell subpopulation. Side scatter (SSC), phycoerythrin (PE), phycoerythrin-cyanine 5 (PE-Cy5). Scale bars: 50 μm (Aa–b), 20 μm (Ac–d).

As the solid epithelial cords canalize, beginning at the urethra and proceeding distally towards the ductal tips, the epithelium reorganizes into two distinct cell populations. Basal epithelial cells are localised along the basement membrane and form a discontinuous layer of cells expressing cytokeratins 5/14 and p63. Instead, tall columnar luminal cells line the ductal lumina and express cytokeratin 8/18 [5]. In the proximal region of the ducts that have begun this cytodifferentiation process, the majority of GFP^+^ cells were localized close to the basement membrane that separate the epithelium from the mesenchyme, whereas scarce columnar secretory epithelium cells expressed GFP (Figure 2Aa–b). Immunohistochemical analysis was performed to define the lineage status of the GFP^+^ cells, which formed between P6 and P10 during this transition from solid to canalized ducts. GFP^+^ basement membrane-localized cells expressed the cytokeratin 5 (Figure 2Ac) and cytokeratin 14 (not shown) basal cell markers but did not express the cytokeratin 8 luminal cell marker (Figure 2Ad).

### GFP^+^ cells are a subpopulation of the Lin^−^ Sca-1^+^ CD49f^+^ prostate epithelial basal/stem cell fraction

Cell surface expression of CD49f (integrin α6) and Sca-1, a marker for stem cells in many tissues [8, 15, 29, 30] revealed three discrete populations on lineage-depleted adult prostate cells, corresponding to Lin^−^Sca-1^−^CD49f^+^ (L-S-C+) luminal cell fraction, Lin^−^Sca-1^+^ CD49f^+^ (L-S+C+) basal/stem cell fraction, and Lin^−^Sca-1^+^ CD49f^−^(L-S+C-) stromal cell fraction [8, 17]. Dissociated single-cell suspensions of P6 prostate tissues were analyzed by flow cytometry. Mature non-prostate cell lineage markers were identified using antibodies against Ter119, CD31, CD45, collectively called Lin. Both GFP^med^ and GFP^high^ cell populations were Lin-negative (Figure 2B). GFP^high^ vSMCs showed no or very little expression of Sca-1 and CD49f cell surface markers (Figure 2C); this result was consistent with those reported earlier in mammary tissue of Tg11.5kb-GFP mice [27]. GFP^med^ epithelial cells appeared as a homogeneous cell population of Sca-1^med^/CD49f^+^ (Figure 2C) with the vast majority of the cells (76.03% ± 0.43, *n* = 3) in the L-S+C+ basal/stem cell fraction and 20.18% ± 0.80 (*n* = 3) in the L-S-C+ luminal cell fraction. Similar results were obtained at P8 and P10, but for later stages, not enough GFP^med^ cells were present to perform proper analysis (Table 1). Further flow cytometry analysis showed that L-S+C+ cell population represented 8.88% ± 3.9 (*n* = 3) of Lin^−^ P6 neonatal cells and that GFP^med^ cells corresponded to a subset of 9.26% ± 2.5 (*n* = 3) of these L-S+C+ cells (Figure 2D). Thus, the vast majority of GFP^med^ epithelial cell fraction is contained into the basal/stem cell L-S+C+ fraction accordingly to their basal localization during cytodifferentiation.

**Table 1:** Quantification of the percentage of prostate GFP^med^ cells at different ages (P6, P8, P10) and their respective distribution in individual cell populations separated by lineage (Lin or L), Sca-1 (S), and CD49f (C) cell surface markers

Gates	P6 (% of Lin-)	P8 (% of Lin-)	P10 (% of Lin-)
L- GFPmed	1.23 ± 0.43	0.70 ± 0.07	0.57 ± 0.12
L-S-C+	20.18 ± 0.80	18.20 ± 5.91	28.52 ± 8.12
L-S+C+	76.03 ± 0.81	65.17 ± 6.51	54.64 ± 7.93
L-S-C-	3.25 ± 1.27	13.78 ± 0.34	14.70 3.42
L-S+C-	0.53 ± 0.41	2.84 ± 1.38	2.14 ± 1.13

Data represent mean values ± s.d. of three independent experiments.

### s-SHIP expression enriches for sphere-forming cells from the mouse neonatal prostate

**Figure 3:**
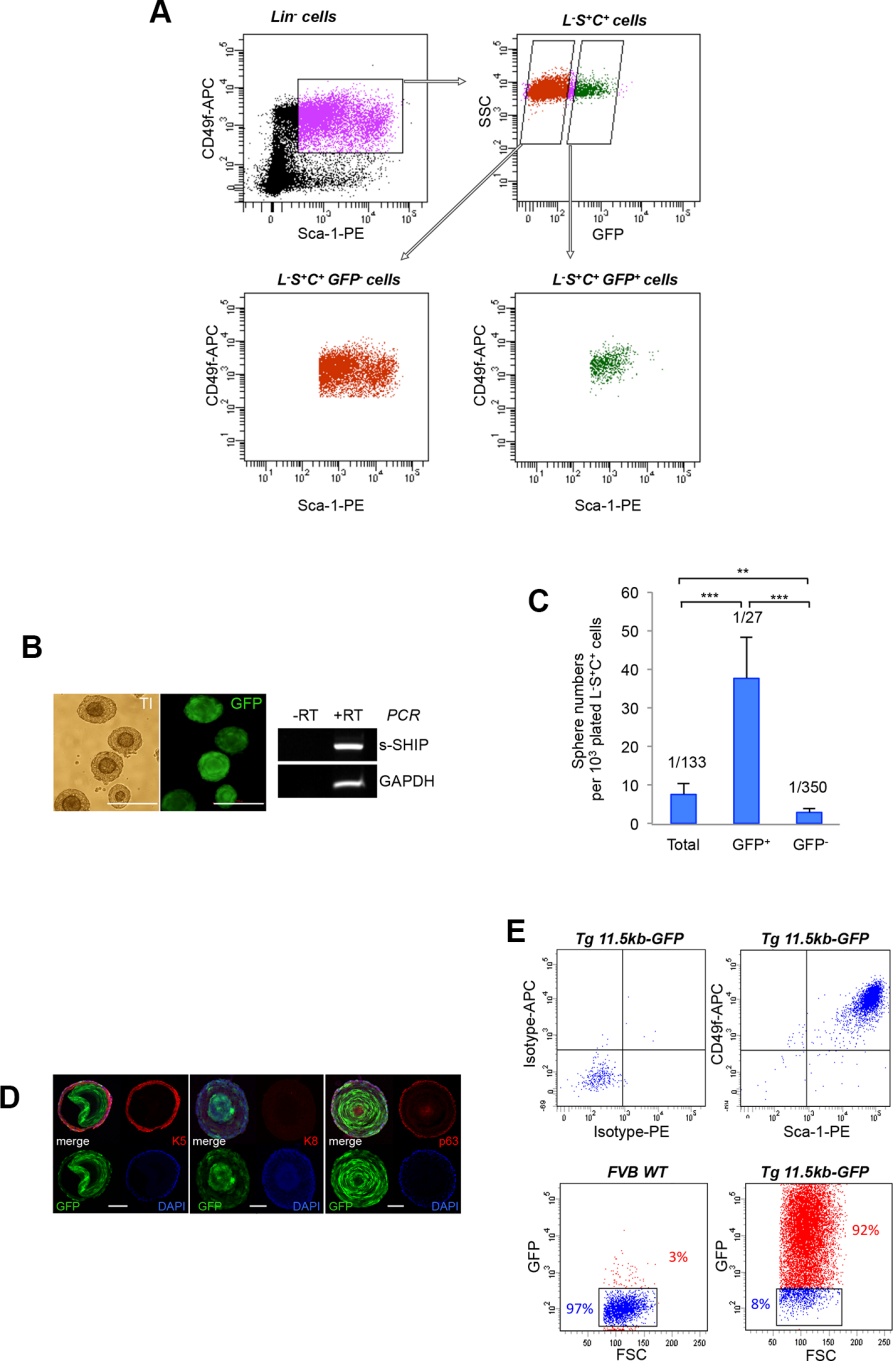
The s-SHIP/GFP-expressing subpopulation is enriched for sphere–forming cells (**A**) Dissociated prostate cells isolated from P6 Tg 11.5kb-GFP mice were stained with V450-conjugated antibodies against non-prostate cell lineage markers (Lin), PE-conjugated antibodies anti-Sca-1 and APC-conjugated antibodies anti-CD49f. Representative FACS plots (*n* > 10) show gates drawn for sequential cell sorting of total Lin^−^ Sca-1^+^ CD49f^+^ (L-S+C+) cells, then L-S+C+ GFP^−^ cells or L-S+C+ GFP^+^ cells. (**B**) Representative images (*n* = 3) of double–layered prostate spheres derived from LSC GFP^+^ cells (left panels); representative RT-PCR analysis (*n* = 2) of s-SHIP transcript expression in sphere cells (right panel). (**C**) Bar graph shows the percentage of sphere-forming cells in total, GFP^+^ or GFP^−^ LSC cell populations that were sorted from prostate single-cell suspension isolated from P6 Tg 11.5kb-GFP mice, plated in Matrigel culture and grown for 10 days. Data represent the mean ± s.d. of three independent experiments, *p* values was determined by Student's test ***p < 0.001, **p < 0.01. (**D**) Immunostaining for basal cell marker cytokeratin 5 (K5) (left panel), luminal cell marker cytokeratin 8 (K8) (middle panel), basal cell marker p63 (right panel) on frozen sections of spheres derived from GFP^+^ LSC cells. (**E**) Sphere cells have a homogenous L-S+C+ GFP^+^ phenotype. Representative flow cytometry analysis (*n* = 3) of CD49f and Sca-1 cell surface markers (upper panels) and GFP (lower panels) expression on dissociated prostate sphere cells. Transillumination (TI), reverse transcription-polymerase chain reaction (RT-PCR), side scatter (SSC), phycoerythrin (PE), allophycocyanin (APC). Scale bars: 250 μm (B), 50 μm (D).

Having confirmed that s-SHIP promoter expression separates the neonatal basal/stem L-S+C+ (thereafter called LSC) cell population into 2 subpopulations, we sought to determine whether GFP^+^ and GFP^−^cells were functionally distinct among the LSC cell population. Sphere-formation in anchorage-independent conditions is a characteristic of tissue stem cells; prostate stem cells grown in Matrigel/PrEGM medium form clonal spheroids exhibiting an organized structure and show self-renew capability on serial passage [18, 21, 31]. From dissociated P6 prostate cells, we first isolated the LSC basal cell population, which contained only CD24^+^ epithelial cells (Supplementary Figure 1A); LSC subset was further fractionated into GFP^+^ and GFP^−^ cell subpopulations (Figure 3A), and tested for spheroid formation. After 7–10 days in culture, solid spheroid structures were observed and displayed a characteristic double–layered appearance with dead cells in the compact core of the spheres as previously described [31] (Figure 3B). In our experimental conditions, the total LSC fraction formed spheres at a rate of 1/133, which is lower to what has been previously reported with adult LSC cells [8] and may be due to technical issue related to tissue dissociation. The LSC GFP^+^ fraction enriched for sphere-forming cells to a frequency of 1/27, a 5-fold enrichment over the total LSC cells, whereas the LSC GFP^−^ fraction only formed spheres at a rate of 1/350 (Figure 3C). Similar results were obtained when we isolated the Lin^−^ CD24^+^ GFP^+^ and Lin^−^ CD24^+^ GFP^−^ epithelial cell populations from dissociated P6 prostate cells (Supplementary Figure 1B) and plated these cells into the sphere assay (Table 2). The sphere-forming capacity was almost totally contained within the Lin^−^ CD24^+^ GFP^+^ fraction. Interestingly, most of sphere cells were GFP^+^ and accordingly they expressed s-SHIP transcript (Figure 3B). Immunostaining of sphere sections showed co-expression of GFP with K5 and p63 basal markers but not with K8 luminal marker (Figure 3D). FACS analysis of dissociated sphere cells (Figure 3E) confirmed GFP expression, and a Sca-1^+^ CD49f^+^ basal cell phenotype as previously described [31]. Spheres were dissociated into single cells that were reseeded to form secondary, and then tertiary spheres (Table 3). For both LSC GFP^−^ and LSC GFP^+^ -derived sphere cell populations, we observed the formation of secondary and tertiary spheres with no significant difference, demonstrating self-renewal of sphere-forming cells in the parental spheres as previously described [31].

**Table 2:** Quantification of the frequency of sphere-forming cells in epithelial Lin^−^ CD24+ cell population separated by the expression of GFP

Populations	Sphere numbers per 10^3^ plated cells	Sphere-forming unit per cell
Total Lin^−^CD24^−^ cells	< 0.05	< 1/20000
Total Lin^−^CD24^+^ cells	0.47 ± 0.19	1/2128
GFP^+^ Lin^−^CD24^+^ cells	17.92 ± 3.00	1/56
GFP^−^ Lin^−^CD24^+^ cells	0.18 ± 0.15	1/5555

Data represent mean values ± s.d. of three independent experiments The sphere number per 10^3^ plated cells obtained with GFP^+^ Lin^−^ CD24^+^ cells was significantly different from those obtained with GFP^−^ Lin^−^ CD24^+^ cells or Total Lin^−^ CD24^+^ cells, with *p* < 0.001 as determined by Student's test.

**Table 3:** Quantification of the number of daughter spheres formed from dissociated primary or secondary sphere cells initially derived from either GFP^+^ LSC cells or GFP^−^ LSC cells

	GFP-positive-derived sphere cells	GFP-negative-derived sphere cells
Secondary	1 sphere for 216 ± 43 plated cells	1 sphere for 188 ± 28 plated cells
Tertiary	1 sphere for 196 ± 29 plated cells	1 sphere for 183 ± 23 plated cells

Data represent mean values ± s. d. of three independent experiments.

### Epithelial GFP^+^ cells give rise to prostatic ducts *in vivo*

**Figure 4:**
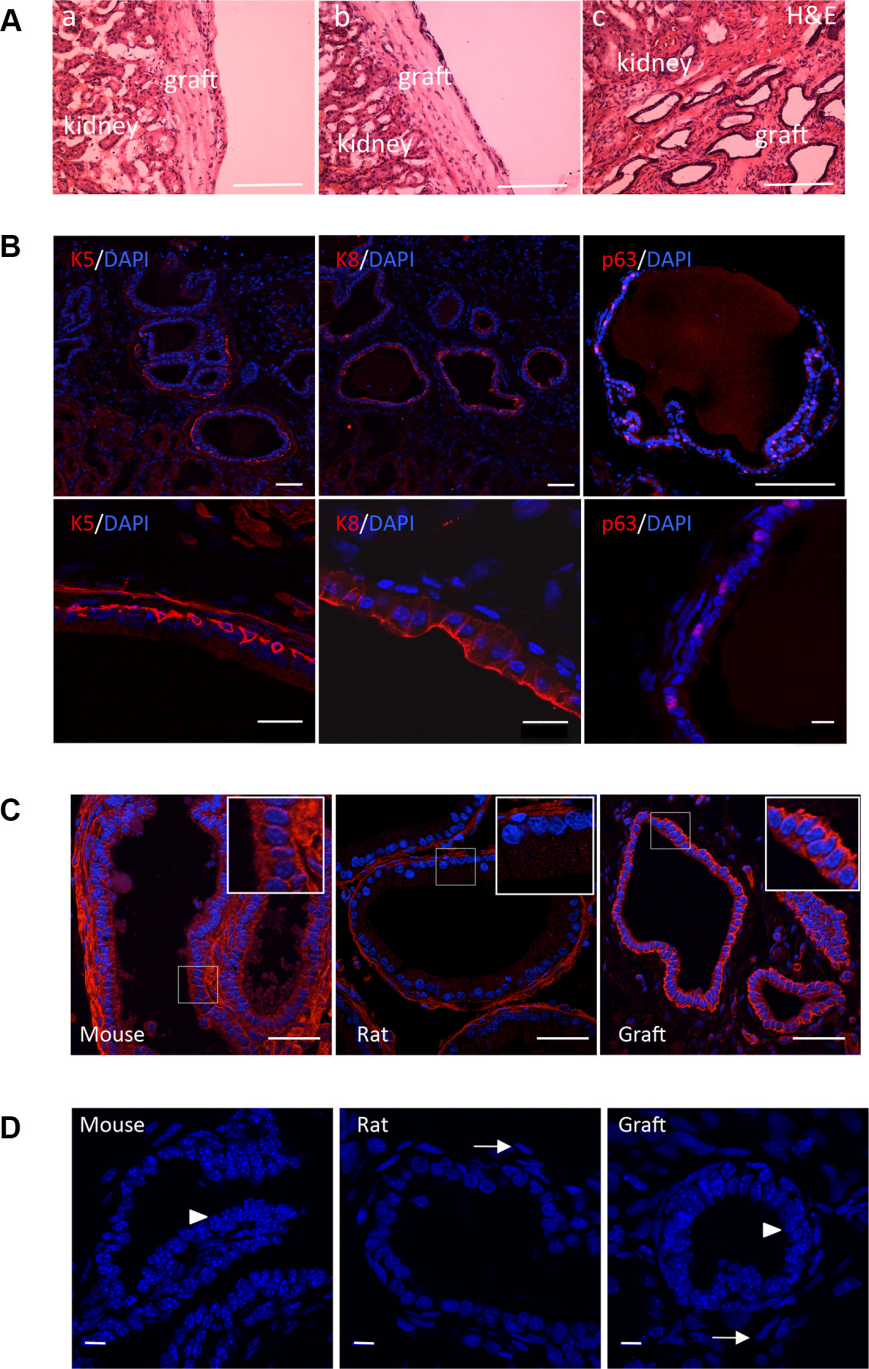
s-SHIP/GFP-expressing cells differentiate *in vivo* to produce mature prostatic tubules containing both basal and luminal cells (**A**) Representative photographs (*n* = 5) showing hematoxylin/eosin (H&E) staining of regenerated tissue sections, eight weeks after grafting UGSM only (a), or UGSM combined with CD24^+^ GFP^−^ (b), or with CD24^+^ GFP^+^ (c) prostate epithelial cells. (**B**) Representative images (*n* = 5) of tissue sections of grafts regenerated from the CD24^+^ GFP^+^ cell population; sections were stained (red) with antibodies against cytokeratin 5 (K5), cytokeratin 8 (K8), and p63. Each tissue section was counterstained with DAPI nuclear stain (blue). Lower panels are regions at higher magnification. (**C**, **D**) The prostate outgrowth tissue is of mouse origin, while the adjacent mesenchymal cells are of rat origin. Representative images (*n* = 5) of tissue sections of grafts regenerated from the CD24^+^ GFP^+^ cell fraction; adult mouse and rat prostate tissue sections were used as positive and negative controls, respectively. Tissue sections were stained with mouse-specific anti β1 integrin antibody (C) and Hoechst dye 33258 (D). Punctate nuclear staining was indicative of murine cells (D, arrowheads) whereas rat cells exhibits a diffuse nuclear staining (D, arrows). Scale bars : 400 μm (A), 100 μm (B, upper panels), 10 μm (B, lower panels), 50 μm (C), 10 μm (D).

Tissue recombination procedures have demonstrated that prostate tissue can be grown *de novo* when fragments of adult rodent prostate tissue are combined with fragments or dissociated cells of the urogenital sinus mesenchyme (UGSM) and implanted under the kidney capsule of immunodeficient mice [32]. UGSM cells expanded by short-term culture also support engraftment of prostate epithelial cells [33]. Single-cell suspensions of rat UGSM were isolated from 18-day-old rat embryo rather than mouse embryo because rat UGSM promotes growth more effectively than does mouse UGSM [7]. Dissociated prostate cells from P6 Tg 11.5-kb-GFP mice were fractionated by FACS between GFP^+^ and GFP^−^ epithelial CD24^+^ cell populations. Next 1,000 cells from each fraction were combined with 200,000 UGSM cells, implanted under kidney capsule of SCID mice and harvested after 8 weeks. In five independent experiments, 8–week-old grafts of UGSM alone or GFP^−^ CD24^+^ cells plus UGSM produced small, opaque, fibrous outgrowths that were negative for prostate epithelial tissue (Figure 4Aa, b). In contrast, all grafts of GFP^+^ CD24^+^ cells combined with UGSM (5 independent experiments) were large, translucent and showed glandular epithelial structures with obvious lumens under microscopic examination (Figure 4Ac). Immunofluorescence analysis demonstrated that these epithelial tubules were composed of basal (K5^+^, p63^+^) and luminal (K8^+^) cells (Figure 4B), thus demonstrating that GFP^+^ CD24^+^ cells were capable of multilineage differentiation. No GFP^+^ cells were detected in these regenerated prostate tissues (not shown). We confirmed that grafts were from mouse origin and not due to contamination of rat epithelial cells from the UGSM stromal cell preparation, using a mouse-specific β1-integrin antibody [15] (Figure 4C) and a typical punctate nuclear staining with the Hoechst dye labeling [13, 29] (Figure 4D). We conclude from these kidney capsule transplantation experiments that GFP^med^ epithelial prostate cells contain the potential for multilineage differentiation to mature prostate epithelium within a ductal structure.

### Androgen-induced prostate regeneration does not induce s-SHIP promoter activation in castrated adult prostate

**Figure 5:**
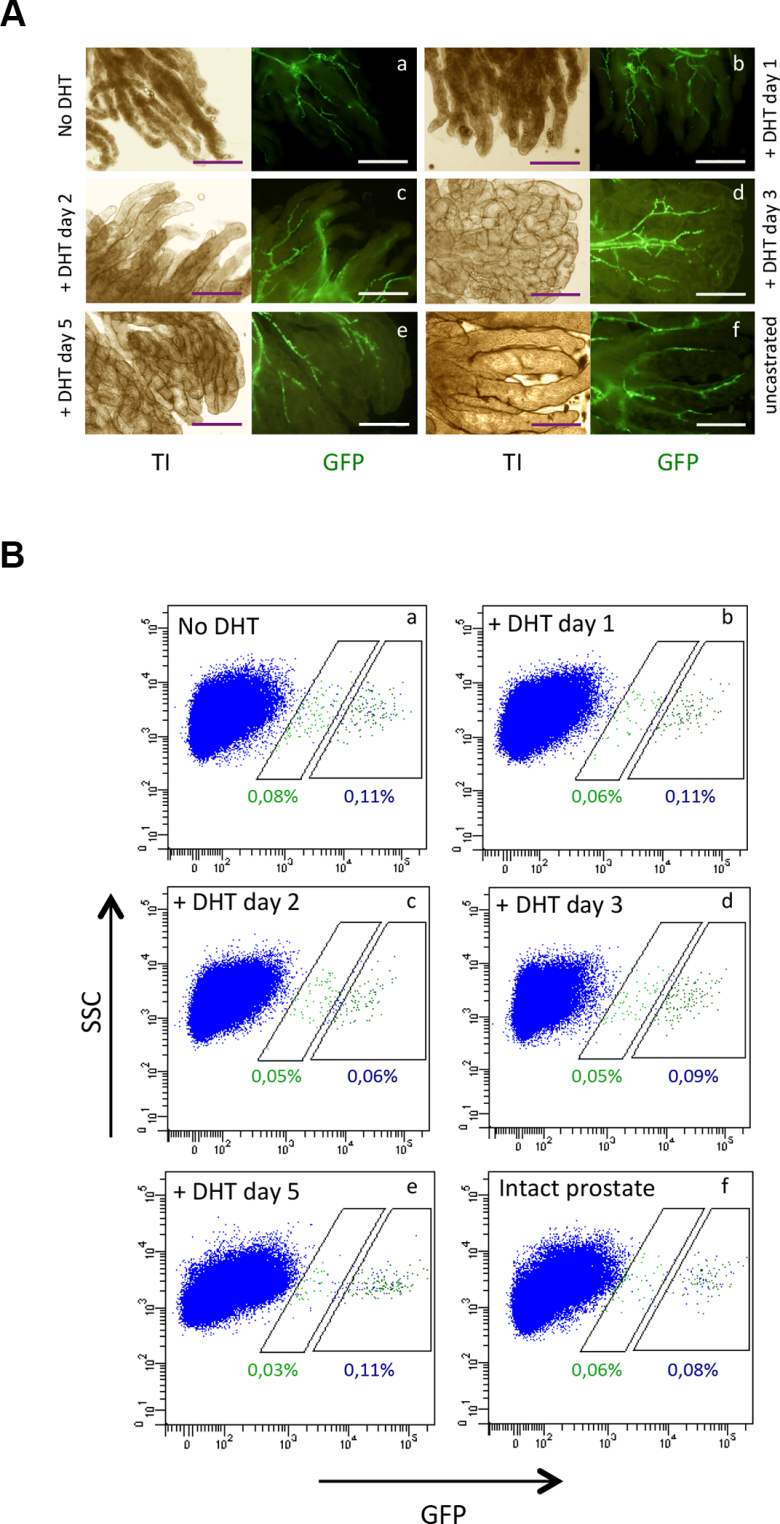
Androgen does not induce s-SHIP promoter expression in adult prostate after four weeks of androgen castration Eight-week-old male Tg11.5kb-GFP mice were castrated by bilateral orchiectomy and after four weeks, prostate regeneration was induced by dihydroxytestosterone (DHT) injection. DHT was injected daily for 1, 2, 3, or 5 days. (**A**) Representative images (*n* = 3) of whole-mount of prostate lobes under a fluorescence microscope. (**B**) Representative flow cytometry analysis of GFP (*n* = 3) expressed by dissociated prostate cells obtained from castrated male mice untreated (a), or treated with DHT for 1 day (b), 2 days (c), 3 days (d), 5 days (e) or from intact uncastrated control mice (f). Transillumination (TI), side scatter (SSC). Scale bars: 250 μm (A).

Since androgens are major regulators of prostate development [5], we investigated s-SHIP promoter expression during early stages of androgen-induced regeneration after castration-induced degeneration. Male mice were castrated to induce prostatic involution, followed four weeks later by androgen replacement to induce prostatic regeneration. Regrowth of the atrophied prostate was rapidly obtained upon androgen treatment but no GFP^+^ cells were observed in the growing ducts and only GFP^+^ vSMCs were detectable (Figure 5A and Supplementary Figure 2). FACS analysis of dissociated cell suspensions confirmed that the percentage of GFP^+^ cells in prostate tissue from castrated mice was not increased after administration of androgen (Figure 5B). These results demonstrate that s-SHIP promoter expression was not reactivated during androgen-induced prostate regeneration in castrated adult mice. Since in adult prostates, both basal cells and luminal cells are independently self-sustained by either unipotent stem/progenitors or by self-duplication [12], our results suggest that s-SHIP promoter expression don't mark these unipotent stem/progenitor cells that mediate adult prostate regeneration.

### Epithelial GFP^+^ cells express genes suggestive of prominent Wnt signaling

**Figure 6:**
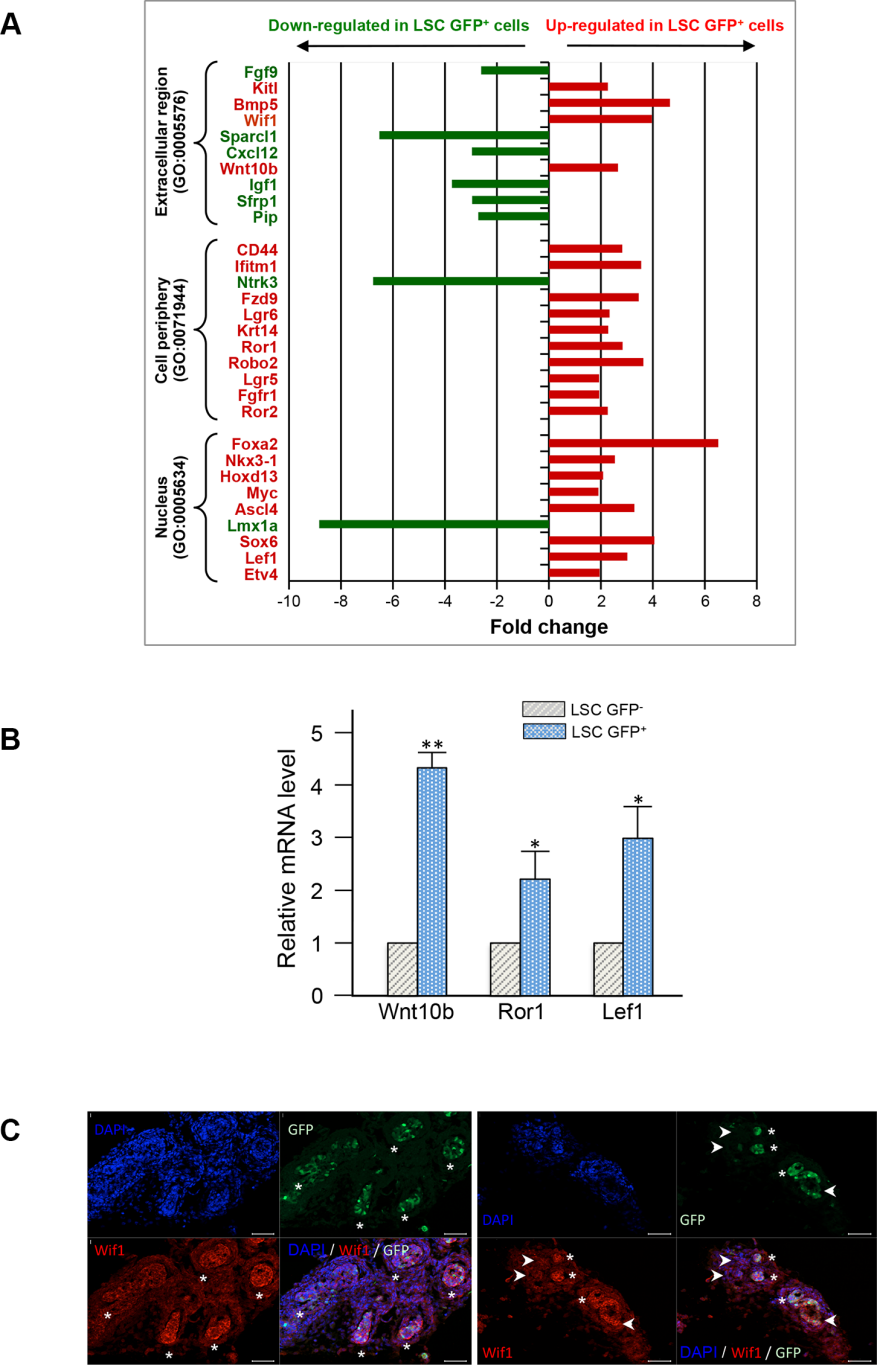
Profiling of gene expression in LSC GFP+ versus LSC GFP^−^ basal cells (**A**) Total RNA was isolated from three independent samples of sorted LSC GFP^+^ and LSC GFP^−^ cells and linear RNA amplification was performed to obtain sufficient amount of high-quality RNA for analysis by SurePrint G3 Mouse GE 8 × 60K microarray (Agilent). Thirty genes were selected from the master list of genes differentially regulated (at least 1.9 fold with a *p* value < 0.05) and grouped into three different Gene Ontology (GO) terms. Up-regulated or down-regulated genes in LSC GFP^+^ cells as compared to LSC GFP^−^ cells are presented in red and green, respectively. (**B**) Real –time quantitative RT-qPCR confirmation of 3 candidate genes between LSC GFP^−^ and LSC GFP^+^ cells. Relative gene expressions were normalized by comparison with the expression of GAPDH and analysed using the 2^−^ ΔΔ ^CT^ method. The expression values were adjusted by setting the expression of LSC GFP^−^ to be 1 for each gene. Data represent means with s.d. from three independent experiments, *p* values was determined by Student's test **p < 0.01, *p < 0.05. (**C**) Representative photographs of immunofluorescent staining (red) of P6 frozen prostate sections for WIF1; white asterisks show GFP^+^ cells expressing Wif1 and white arrows-heads mark GFP^−^ cells expressing a lower level of Wif1. Sections were counterstained with DAPI nuclear stain (blue). Scale bars: 50 μm (C).

We performed transcriptional profiling of LSC GFP^+^ vs LSC GFP^−^ cells to distinguish a stem cell from a basal cell profile using Agilent microarrays technology based on 55,681 probes. After probes filtration, transcriptional levels of 37,023 probes were analyzed among which 2,819 differentially expressed transcripts between the two cell populations (http://www.ncbi.nlm.nih.gov/geo/query/acc.cgi?acc=GSE73758). They corresponded to 2,090 Entrez Gene IDs that were mapped to 1,878 unique Entrez Gene IDs being differentially expressed (*p*-value < 0.05) between LSC GFP^+^ and LSC GFP^−^cells. Among these, 1,121 probes corresponding to 999 transcripts whose expression was statistically up- or down-regulated ≥ 1.5 fold in the LSC GFP^+^ cells vs the LSC GFP^−^cells. Analysis based upon the 513 up-regulated genes in LSC GFP^+^ cells reveals the enrichment on different biological processes including morphogenesis of a branching epithelium (GO:0061138), gland morphogenesis (GO:0022612) and Wnt receptor signaling pathway (GO:0016055). Especially, LSC GFP^+^ subpopulation was enriched for transcripts of basal cell markers like *Krt5*, *Krt14*, *Trp63*, pluripotency regulators like *Sox2*, *Klf4*, *Lef1*, *My*c, and basement membrane components and their receptors like *Col4a1*, *Col4a2*, *Itga3*, *Sdc1*. Analysis based upon the 486 down-regulated genes in LSC GFP^+^ cells did not highlight any peculiar biologic pathway but contained several interesting genes in the context of stem cells. Thirty genes related to stem cell biology that displayed a fold change in expression higher than 1.9 were selected (Figure 6A). Foremost, among these 30 genes, a number of Wnt pathway members were indentified, including components implicated in both Wnt/β-catenin-dependent (canonical) or non-canonical signaling such as *Wnt10b, Lgr5, Lgr6, Fzd9*, *Ror 1, Ror 2*, and corresponding to Wnt target genes like *c-Myc*, *Lef1*. This expression pattern fits well with the fundamental role of Wnt pathway in different adult stem cells, including PSCs [34–37]. LSC GFP^+^ cells also differentially expressed genes that modulate Wnt pathway activity, with down-regulation of secreted frizzled-related *Sfrp1* and upregulation of Wnt inhibitory factor *wif1* transcripts, suggesting a tight control of these pathways in LSC GFP^+^ cells and a possible regulation of neighboring cells. Out of these, 3 genes were selected for validation of the microarray results by RT-qPCR. As shown in Figure 6B, RT-qPCR analysis confirmed the microarray data with Wnt10b, Ror1, and Lef1 genes found to be similarly up-regulated by both methods. In P6 Tg 11.5kb-GFP prostate section, immunostaining of Wif1 also confirmed that GFP^+^ cells showed more intense staining (Figure 6C, asterisks) than GFP^−^ cells (Figure 6C, arrow-heads) in the prostatic buds.

## DISCUSSION

Prostate epithelial cells that express the s-SHIP promoter were only observed during a limited time period, around two weeks after birth. At that time, branching morphogenesis is almost entirely complete, suggesting that s-SHIP/GFP-expressing cells may contribute to the early formation of the prostate cellular architecture. Serum testosterone levels are low during this time; then puberty starts with testosterone levels rising significantly, and prostatic growth increases more rapidly than during the early postnatal period [5]. The reciprocal expression of androgen and GFP in prostate cells suggests that s-SHIP promoter expression is minimally dependent of androgen; accordingly, we could not detected any s-SHIP promoter reactivation during regeneration induced by testosterone after castration-induced prostate involution. This result is consistent with the K5/14^+^ LSC basal phenotype of GFP^+^ cells. Numerous studies favor the hypothesis that basal cells contains the prostate stem cell activity [8, 15–18, 24, 29, 31, 33, 38]. However, lineage-tracing experiments have generated evidence that stem populations could be localized in the luminal layer [12–14, 39]. A 3D culture method supporting long-term expansion of primary mouse and human organoids [40] confirmed that in adult prostate, epithelia could be maintained by respective progenitor/stem within the basal and luminal cell lineages [41]. Concerning the neonatal prostate, Ousset and colleagues demonstrated that cells possessing the multilineage differentiation capacity belong to the basal lineage, while luminal cells have committed to become unipotent [10, 11, 42]. Moreover, during early prostate development, ΔNp63-positive basal cells represent the multipotent progenitor/stem cell population [43]. Altogether, these studies imply a developmental stage-specific switch of the mechanisms for prostate epithelial maintenance [41, 42]. Based on the data presented here, we propose that s-SHIP promoter expression marks a subset of basal cells corresponding to these multipotent neonatal murine stem cells.

Prostasphere culture is a useful method to identify and propagate prostate stem cells. Although the most majority of sphere cells presents an homogeneous expression of tissue prostate stem cells markers, L-S+C+ [31] and s-SHIP/GFP (Figure 3), only a few of them are capable of forming daughter spheres *in vitro* [31] (Table 3) and regenerate glandular structures *in vivo* [21, 31]. Therefore, additional markers remains to be identified in order to isolate this peculiar sphere cell subpopulation. Interestingly, rare long-term label retaining cells have been recently described in prostaspheres and these slow-cycling cells possess high capacity to form prostaspheres [44].

We have previously shown that s-SHIP-GFP promoter reporter tracks subset of human prostate RWPE-1 cells enriched in stem cell characteristics [45]. s-SHIP is also expressed in numerous cancer cell lines and xenograft–maintained human prostate cancers (Supplementary Figure 3A) which represent relevant models of prostate cancer [46–48]. Transfection of s-SHIP-GFP promoter reporter in human cancer cells showed that they were heterogeneous in term of s-SHIP promoter expression (Supplementary Figure 3B), like for other stem cell markers [49]. Cancer stem cells are present in prostate cancer cell lines [49]; more experiments are now needed to determine the relationship between these cancer stem cells and s-SHIP-expressing cells.

s-SHIP protein is a shorter isoform of SHIP1 (SH2-containing Inositol 5ʹ-Phosphatase) protein that lacks the N-terminal domain region [25]. Initially, a special feature of s-SHIP isoform was its specific expression in embryonic stem cells and in primitive hematopoietic stem cells, but not in their more mature progeny [25, 50]. The cloning of the s-SHIP promoter region enabled isolation and analysis of mammary stem cells [27, 51–54], but the role of s-SHIP protein remains unknown. Its ubiquitous homologue SHIP2 is crucial for maintaining breast cancer stem cells [55]. Thus, s-SHIP may play a role, for example in modulating the PI3K/AKT pathway in prostate cancer stem cells [56]. The SHIP specific inhibitor (SHIPi) [57, 58] should offer a valuable opportunity to address this issue.

We performed transcriptional profiling to distinguish a tissue stem cell profile (LSC GFP^+^ cells) from a basal cell profile (LSC GFP^−^ cells). In GFP^+^ LSC cells, we observed an increased expression of transcriptional regulators of early prostate development, such as Hoxd13, Nkx3.1 and FoxA2 [59, 60]. Similarly to s-SHIP, FoxA2 is expressed during the early stages of prostate bud formation whereas its expression is restricted to rare basal epithelial cells within the periurethral ducts in adult prostate [61]. Based on its role in prostate cancer [62], FoxA2 may be associated with the invasive property of neonatal stem cells. Increased expression of genes associated with various signaling pathways was observed in the LSC GFP^+^ population, such as *Bmp5, kitl*. However, the most represented components belong to the Wnt signaling pathway that is known to plays a fundamental role in multiple adult stem cells [34–37], as well as in orchestrating proper prostate gland development and maintenance [63, 64]. Wnt signaling is more active at the early stages of developing prostates but rapidly decline as the prostate matured [65] around the same time that GFP expression is lost. Our results are also in accordance with the study of Roarty and coworkers showing that s-SHIP/GFP expression mirrored the expression gradient and localization of Wnt/β-catenin activity in terminal end buds of the mammary gland in Tg11.5kb-GFP mice [54]. It is also worth noting the upregulation of Ascl4 [66]. Its function is unknown but its homolog Ascl1 plays a role in neural stem cells and interacts with Wnt signaling [67, 68].

Considering down-regulated genes in LSC GFP^+^ cells, expression level of several of them inversely correlates with cancer aggressiveness, notably in prostate such as SPARCL1, a negative regulator of bud expansion and prostasphere growth [69, 70], and SFRP1, a negative regulator of Wnt pathway [71]. Lmx1a is methylation-silenced in various carcinoma [72–74] and could act as a suppressor of metastasis, invasion and epithelial-mesenchymal transition in cancer cells [72–74]. Finally, Ntrk3 is a potential tumor suppressor inactivated in colon cancer [75] and acts as a dependence receptor [76], therefore its down-regulation in LSC GFP^+^ cells might create a clonal survival advantage in the absence of its ligand NT-3.

In conclusion, a unique feature of this study was the ability to isolate PSCs using s-SHIP promoter driven GFP as a single marker. While the extensive proliferation, migration, and invasion required for early prostate development do not occur in the resting adult prostate, they do resemble processes mediating prostate cancer progression [57, 77, 78]. Thus, gene expression of these neonatal prostate stem cells may reveal new targets to aid detection, prognosis and treatment of prostate cancer.

## MATERIALS AND METHODS

### Animals and tissue collection

FVB/N transgenic (Tg) 11.5kb-GFP, wild–type FVB/N, CB17 SCID mouse strains and Sprague Dawley rats (Charles River Laboratories, Wilmington, MA) were housed and bred in accordance with institutional guidelines for humane animal treatment and all animal studies were conducted in accordance with Institutional Animal Care and Use Committee approved protocols. Castration was performed on 8 week-old male mice by bilateral orchiectomy using standard techniques, with the fully regressed state attained at 4 weeks after castration. For prostate regeneration by androgen replacement, testosterone (Sigma, St Louis, MO; A8380) was dissolved at 25 mg/ml in 100% ethanol, diluted in phosphate buffer saline (PBS, Gibco, ThermoFisher Scientific, Waltham, MA) to a final concentration of 2.5 mg/ml and 100 μl were injected by intraperitoneal injection once a day for 1 to 5 consecutive days. Prostate tissue was collected from mice, minced into small fragment, digested with 200 U/ml collagenase IA–S (Sigma; C5894) in Dulbecco's modified Eagles medium (DME, Gibco) supplemented with 10% fetal bovine serum (FBS, Hyclone, South Logan, UT) (DME-10% FBS) at 37°C for 60 min with gentle agitation. The digested cells were filtered through a 40-μm cell strainer (BD Biosciences, Franklin Lakes, NJ), washed, and resuspended in DME-10% FBS.

### Immunofluorescence and histological analysis

Tissues sections were obtained and analysed as previously described [27]. The following antibodies were used: cytokeratin 5 (1:500, PRB-160P; BioLegend, San Diego, CA), cytokeratin 14 (1:500, PRB-155P; BioLegend), cytokeratin 8 (1:500, MMS-162P; Biolegend), p63 (1:100, sc-8431, clone 4A4; Santa Cruz Biotechnologies, Santa Cruz, CA), integrin β1 (10 mg/ml, MAB1997, clone MB1.2, Chemicon, Temecula, CA), Alexa Fluor 594 phalloidin (1:100, Molecular Probes, Eugene, OR), alpha smooth muscle actin–cy3 (1:400, C6198, Sigma), Wif1 (1:100, ab33281, abcam, Paris, France). Secondary antibodies were from Molecular Probe: Alexa Fluor 594 F(ab’)2 fragment of goat anti-rabbit IgG (1:1000, A11072), Alexa Fluor 594 F(ab’)2 fragment of goat anti-mouse IgG (1:1000, A11020), Alexa Fluor 594 F(ab’)2 fragment of goat anti-Rat IgG (1:1000, A11007). Pictures were taken either on a LSM 510 META Confocal Microscope or an Axioplan2 with ApoTome equipment (Carl Zeiss, Germany).

### Fluorescence–activated cell sorting and analysis

Dissociated prostate cells were suspended in DME-10% FBS, preincubated with purified rat anti-mouse CD16/CD32 (mouse BD Fc Block) antibody (1:50, BD Pharmingen, San Jose, CA) at 4°C for 10 min, and stained with specific antibodies at 4°C for 30 min. Flow cytometry analysis was performed using a BD FACS Canto II and analysed by NovoExpress^™^ software (ACEA Biosciences, San Diego, CA). Cell sorting was conducted on a BD FACS Vantage or a BD FACS Aria. Primary antibodies used were: PE-Cy5 rat anti-human/mouse CD49f (1:100, BD Pharmingen), APC rat anti-human/mouse CD49f (1:100, BD Pharmingen), APC rat anti-mouse CD24 (1:100, BD Pharmingen), PE rat anti-mouse Sca-1 (1:100, eBioscience, San Diego, CA). Isotype controls used were from BD Pharmingen : PE rat IgG (1:100), PE-Cy5 Rat IgG (1:100), APC Rat IgG (1 :100). For lineage staining, biotinylated antibodies used were : rat anti-mouse CD31 (1:100, BD Pharmingen), rat anti-mouse CD45 (1:100, eBioscience), rat anti-mouse TER-119 (1:100, eBioscience). Dynabeads M-280 Streptavidin (Invitrogen) or BD Horizon™ V450-Streptavidin (1 :10000, BD Biosciences, Franklin Lakes, NJ) were used.

### *In vitro* prostate sphere–forming assays

Dissociated prostate cells were labeled using antibodies as specified in the text, sorted by FACS, counted, and suspended in 1:1 Matrigel (BD Biosciences)/prostate epithelial growth medium (PrEGM) (Lonza, Walkersville, MD) in a total volume of 100 μl. Samples were plated in a 6–well plate (Costar) and allowed to solidify at 37°C for 20 min, before 3 ml of PrEGM was added. Medium was replaced every 3-4 days; spheres with a double–layered appearance and a diameter >40 μm were counted at day 10, whereas small or more lucent spheroids were not counted according to the original description of the prostate sphere culture technique [31]. To passage 7 day-old spheres, medium was aspirated, and Matrigel was digested by 1 mg/ml of dispase (STEMCELL Technologies Inc, Vancouver BC, Canada) at 37°C for 30 min. Spheres were collected, pelleted, resuspended and digested by 0.05% Trypsin/EDTA at room temperature for 5 min. Dissociated cells were passed through a 40-μm cell strainer, counted and replated.

### *In vivo* prostate regeneration

Mouse prostate regeneration was adapted from previous reports [18, 29, 33]. Briefly, urogenital sinus (UGS) mesenchyme cells were microdissected from day-19 rat embryos and digested with 1 ml of 1% trypsin at 4°C for 30 min with care to avoid over–digestion. UGS were then washed with DME-10% FBS and epithelial tubes were microscopically removed from the opaque mesenchymal tissue using fine forceps and a needlepoint. The mesenchymal tissue (UGSM) was digested with 200 U/ml collagenase IA–S in DME-10% FBS at 37°C for 45 min with gentle agitation. Single cells were passed by filtration through a 40-μm cell strainer and washed with DME-10% FBS. UGSM cells were then cultured in Bfs media [33] for one or two days. Adherent cells were used for transplantation or frozen in liquid nitrogen. For transplantation experiments, 2 × 10^5^ UGSM cells were mixed with dissociated 10^3^ FACS-isolated prostate cells in 10 μl of DME-10% FBS and embedded in 20 μl cold rat–tail collagen high concentration (BD Biosciences) that was neutralized before use with NaOH. Collagen was plated as “buttons” onto a culture dish at 37°C. After collagen polymerization, buttons were implanted under kidney capsules of male SCID mice, and a testosterone pellet (25.0 mg/pellet, 60–day release; Innovative Research of America, Sarasota, FL) was s.c. implanted at the same time. Two month later, animals were euthanized and their kidneys were harvested. The portion of the kidneys containing the grafts was fixed, embedded in OCT, sectioned, and stained. All surgeries were performed in animal facilities using protocol approved by the FHCRC Animal Care and Use Committee.

### Quantitative RT–PCR

Total RNA was extracted using an RNeasy Micro purification kit (Qiagen, Courtaboeuf, France) and between 0.1 and 1 μg of total RNA was reverse transcribed using Quantitect reverse transcriptase kit (Qiagen). One-tenth of the first-strand cDNA synthesis product was used as template for the qRT–PCR reactions by using SYBR green Master Mix (Eurogentec, Angers, France) on a MX3005MP Stratagene cycler. Primers used have been previously described for mouse [27] and human [45] s-SHIP. Other primers are listed in the Supplementary Material List 1. The thermal cycling program was 95°C for 20 sec, followed by a hybridization step at 62°C for 30 sec, and an elongation step at 72°C for 45 sec.

### Microarrays experiments

Total RNA was extracted with the RNeasy Plus Micro Kit (Qiagen) and was quantified with the NanoDrop ND-1000 (ThermoFisher Scientific). Quality of the extracted RNA was evaluated with the Agilent 2100 Bioanalyzer system (Agilent Technologies, Massy, France). Forty nanograms of total RNA per sample was amplified with the Ovation^®^ Pico WTA System Kit (NuGEN, San Carlos, CA), then two micrograms of amplified and purified cDNA was labeled with the SureTag DNA Labeling kit (Agilent Technologies). Cy3-labeled cDNA samples (2.5 μg) were used for 17-hour hybridization at 65°C to the SurePrint G3 Mouse GE 8 × 60K Microarrays (Agilent Technologies). All procedures were carried out according to the manufacturer's instructions. The hybridized microarrays were scanned with the Agilent DNA Microarray Scanner (G2505C). Signal intensity was quantified from the scanned image by using Feature Extraction software version 11.0.1.1 (Agilent Technologies). These experiments were performed by Imaxio (Clermont-Ferrand, France). The data discussed in this publication have been deposited in NCBI's Gene Expression Omnibus [79] and are accessible through GEO Series accession number GSE73758 (http://www.ncbi.nlm.nih.gov/geo/query/acc.cgi?acc=GSE73758).

### Statistical analysis

Data are expressed as mean values ± standard deviation (s.d.) of at least 3 independent experiments. The statistical analysis was done by using Student's *t*-test and *p* value < 0.05 was considered significant.

## SUPPLEMENTARY MATERIALS FIGURES



## References

[1] He S, Nakada D, Morrison SJ (2009). Mechanisms of stem cell self–renewal. Annu Rev Cell Dev Biol.

[2] Li L, Clevers H (2010). Coexistence of quiescent and active adult stem cells in mammals. Science.

[3] Visvader JE (2011). Cells of origin in cancer. Nature.

[4] Matchett KB, Lappin TR (2014). Concise reviews: cancer stem cells: from concept to cure. Stem Cells.

[5] Marker PC, Donjacour AA, Dahiya R, Cunha GR (2003). Hormonal, cellular, and molecular control of prostatic development. Dev Biol.

[6] English HF, Santen RJ, Isaacs JT (1987). Response of glandular versus basal rat ventral prostatic epithelial cells to androgen withdrawal and replacement. Prostate.

[7] Goto K, Salm SN, Coetzee S, Xiong X, Burger PE, Shapiro E, Lepor H, Moscatelli D, Wilson EL (2006). Proximal prostatic stem cells are programmed to regenerate a proximal–distal ductal axis. Stem cells.

[8] Lawson DA, Xin L, Lukacs RU, Cheng D, Witte ON (2007). Isolation and functional characterization of murine prostate stem cells. Proc Natl Acad Sci USA.

[9] Tsujimura A, Koikawa Y, Salm S, Takao T, Coetzee S, Moscatelli D, Shapiro E, Lepor H, Sun TT, Wilson EL (2002). Proximal location of mouse prostate epithelial stem cells: a model of prostatic homeostasis. J Cell Biol.

[10] Goldstein AS, Witte ON (2012). A plethora of progenitors in the post-natal prostate. EMBO Rep.

[11] Ousset M, Van Keymeulen A, Bouvencourt G, Sharma N, Achouri Y, Simons BD, Blanpain C (2012). Multipotent and unipotent progenitors contribute to prostate postnatal development. Nat Cell Biol.

[12] Choi N, Zhang B, Zhang L, Ittmann M, Xin L (2012). Adult murine prostate basal and luminal cells are self-sustained lineages that can both serve as targets for prostate cancer initiation. Cancer Cell.

[13] Wang X, Kruithof-de Julio M, Economides KD, Walker D, Yu H, Halili MV, Hu YP, Price SM, Abate-Shen C, Shen MM (2009). A luminal epithelial stem cell that is a cell of origin for prostate cancer. Nature.

[14] Wang ZA, Mitrofanova A, Bergren SK, Abate-Shen C, Cardiff RD, Califano A, Shen MM (2013). Lineage analysis of basal epithelial cells reveals their unexpected plasticity and supports a cell-of-origin model for prostate cancer heterogeneity. Nat Cell Biol.

[15] Leong KG, Wang BE, Johnson L, Gao WQ (2008). Generation of a prostate from a single adult stem cell. Nature.

[16] Goldstein AS, Lawson DA, Cheng D, Sun W, Garraway IP, Witte ON (2008). Trop2 identifies a subpopulation of murine and human prostate basal cells with stem cell characteristics. Proc Natl Acad Sci USA.

[17] Lawson DA, Zong Y, Memarzadeh S, Xin L, Huang J, Witte ON (2010). Basal epithelial stem cells are efficient targets for prostate cancer initiation. Proc Natl Acad Sci USA.

[18] Lukacs RU, Goldstein AS, Lawson DA, Cheng D, Witte ON (2010). Isolation, cultivation and characterization of adult murine prostate stem cells. Nat Protoc.

[19] Collins AT, Habib FK, Maitland NJ, Neal DE (2001). Identification and isolation of human prostate epithelial stem cells based on alpha(2)beta(1)–integrin expression. J Cell Sci.

[20] Richardson GD, Robson CN, Lang SH, Neal DE, Maitland NJ, Collins AT (2004). CD133, a novel marker for human prostatic epithelial stem cells. J Cell Sci.

[21] Garraway IP, Sun W, Tran CP, Perner S, Zhang B, Goldstein AS, Hahm SA, Haider M, Head CS, Reiter RE, Rubin MA, Witte ON (2010). Human prostate sphere–forming cells represent a subset of basal epithelial cells capable of glandular regeneration *in vivo*. Prostate.

[22] Jiao J, Hindoyan A, Wang S, Tran LM, Goldstein AS, Lawson D, Chen D, Li Y, Guo C, Zhang B, Fazil L, Gleave M, Witte ON (2012). Identification of CD166 as a surface marker for enriching prostate stem/progenitor and cancer initiating cells. PLoS ONE.

[23] Goldstein AS, Huang J, Guo C, Garraway IP, Witte ON (2010). Identification of a cell of origin for human prostate cancer. Science.

[24] Goldstein AS, Drake JM, Burnes DL, Finley DS, Zhang H, Reiter RE, J Huang J, Witte ON (2011). Purification and direct transformation of epithelial progenitor cells from primary human prostate. Nat Protoc.

[25] Tu Z, Ninos JM, Ma Z, Wang JW, Lemos MP, Desponts C, Ghansah T, Howson JM, Kerr WG (2001). Embryonic and hematopoietic stem cells express a novel SH2–containing inositol phosphatase isoform that partners with the Grb2 adapter protein. Blood.

[26] Rohrschneider LR, Custodio JM, Anderson TA, Miller CP, Gu H (2005). The intron 5/6 promoter region of the ship1 gene regulates expression in stem/progenitor cells of the mouse embryo. Dev Biol.

[27] Bai L, Rohrschneider LR (2010). s-SHIP promoter expression marks activated stem cells in developping mouse mammary tissue. Genes Dev.

[28] Sugimura Y, Cunha GR, Donjacour AA (1986). Morphogenesis of ductal networks in the mouse prostate. Biol Reprod.

[29] Barclay WW, Axanova LS, Chen W, Romero L, Maund SL, Soker S, Lees CJ, Cramer SD (2008). Characterization of adult prostatic progenitor/stem cells exhibiting self-renewal and multilineage differentiation. Stem cells.

[30] Burger PE, Xiong X, Coetzee S, Salm SN, Moscatelli D, Goto K, Wilson EL (2005). Sca-1 expression identifies stem cells in the proximal region of prostatic ducts with high capacity to reconstitute prostatic tissue. Proc Natl Acad Sci USA.

[31] Xin L, Lukacs RU, Lawson DA, Cheng D, Witte ON (2007). Self–renewal and multilineage differentiation *in vitro* from murine prostate stem cells. Stem cells.

[32] Cunha GR, Lung B (1978). The possible influence of temporal factors in androgenic responsiveness of urogenital tissue recombinants from wild–type and androgen–insensitive (Tfm) mice. J Exp Zool.

[33] Xin L, Ide H, Kim Y, Dubey P, Witte ON (2003). *in vivo* regeneration of murine prostate from dissociated cell populations of postnatal epithelia and urogenital sinus mesenchyme. Proc Natl Acad Sci USA.

[34] Angers S, Moon RT (2009). Proximal events in Wnt signal transduction. Nat Rev Mol Cell Biol.

[35] Holland JD, Klaus A, Garratt AN, Birchmeier W (2013). Wnt signaling in stem and cancer stem cells. Curr Opin Cell Biol.

[36] Lukacs RU, Memarzadeh S, Wu H, Witte ON (2010). Bmi-1 is a crucial regulator of prostate stem cell self-renewal and malignant transformation. Cell Stem Cell.

[37] Luo W, Rodriguez M, Valdez JM, Zhu X, Tan K, Li D, Siwko S, Xin L, Liu M (2013). Lgr4 is a key regulator of prostate development and prostate stem cell differentiation. Stem cells.

[38] Shi X, Gipp J, Dries M, Bushman W (2014). Prostate progenitor cells proliferate in response to castration. Stem Cell Res.

[39] Liu J, Pascal LE, Isharwal S, Metzger D, Ramos Garcia R, Pilch J, Kasper S, Williams K, Basse PH, Nelson JB, Chambon P, Wang Z (2011). Regenerated luminal epithelial cells are derived from preexisting luminal epithelial cells in adult mouse prostate. Mol Endocrinol.

[40] Karthaus WR, Iaquinta PJ, Drost J, Gracanin A, van Boxtel R, Wongvipat J, Dowling CM, Gao D, Begthel H, Sachs N, Vries RG, Cuppen E, Chen Y (2014). Identification of multipotent luminal progenitor cells in human prostate organoid cultures. Cell.

[41] Kwon OJ, Xin L (2014). Prostate epithelial stem and progenitor cells. Am J Clin Exp Urol.

[42] Xin L (2013). A developmental stage-dependent switch of the mechanisms for prostate epithelial maintenance. Asian J Androl.

[43] Pignon JC, Grisanzio C, Geng Y, Song J, Shivdasani RA, Signoretti S (2013). p63-expressing cells are the stem cells of developing prostate, bladder, and colorectal epithelia. Proc Natl Acad Sci USA.

[44] Huang Y, Hamana T, Liu J, Wang C, An L, You P, Chang JYF, Xu J, McKeehan WL, Wang F (2015). Prostate sphere-forming stem cells are derived from the p63-expressing basal compartment. J Biol Chem.

[45] Bauderlique-Le Roy H, Vennin C, Brocqueville G, Spruyt N, Adriaenssens E, Bourette RP (2015). Enrichment of human stem-like prostate cells with s-SHIP promoter activity uncovers a role in stemness for the long noncoding RNA H19. Stem Cells Dev.

[46] Ellis WJ, Vessella RL, Buhler KR, Bladou F, True LD, Bigler SA, Curtis D, Lange PH (1996). Characterization of a novel androgen-sensitive, prostate specific antigen-producing prostatic carcinoma xenograft: LuCaP 23. Clin Cancer Res.

[47] Pascal LE, Vêncio RZ, Vessella RL, Ware CB, Vêncio EF, Denyer G, Liu AY (2011). Lineage relationship of prostate cancer cell types based on gene expression. BMC Med Genomics.

[48] Fontana L, Adelaiye RM, Rastelli AL, Miles KM, Ciamporcero E, Longo VD, Nguyen H, Vessella R, Pili R (2013). Dietary protein restriction inhibits tumor growth in human xenograft models of prostate and breast cancer. Oncotarget.

[49] Miki J, Rhim JS (2008). Prostate cell cultures as *in vitro* models for the study of normal stem cells and cancer stem cells. Prostate Cancer Prostatic Dis.

[50] Desponts C, Ninos JM, Kerr WG (2006). s-SHIP associates with receptor complexes essential for pluripotent stem cell growth and survival. Stem Cells Dev.

[51] Huo Y, Macara IG (2014). The Par3-like polarity protein Par3L is essential for mammary stem cell maintenance. Nature Cell Biol.

[52] Kogata N, Zvelebil M, Howard BA (2013). Neuregulin 3 and erbb signalling network in embryonic mammary gland development. J Mammary Gland Biol Neoplasia.

[53] Kogata N, Oliemuller E, Wansbury O, Howard BA (2014). Neuregulin-3 regulates epithelial progenitor cell positioning and specifies mammary phenotype. Stem Cells Dev.

[54] Roarty K, Shore AN, Creighton CJ, Rosen JM (2015). Ror2 regulates branching, differentiation, and actin-cytoskeletal dynamics within the mammary epithelium. J Cell Biol.

[55] Fu CH, Lin RJ, Yu J, Chang WW, Liao GS, Chang WY, Tseng LM, Tsai YF, Yu JC, Yu AL (2014). A novel oncogenic role of inositol phosphatase SHIP2 in ER-negative breast cancer stem cells: involvement of JNK/vimentin activation. Stem Cells.

[56] Rybak AP, Bristow RG, Kapoor A (2015). Prostate cancer stem cells: deciphering the origins and pathways involved in prostate tumorigenesis and aggression. Oncotarget.

[57] Brooks R, Lyer S, Akada H, Neelam S, Russo CM, Chisholm JD, Kerr WG (2015). Coordinate expansion of murine hematopoietic and mesenchymal stem cell compartments by SHIPi. Stem Cells.

[58] Fuhler GM, Brooks R, Toms B, Lyer S, Gengo EA, Park MY, Gumbleton M, Viernes DR, Chisholm JD, Kerr WG (2012). Therapeutic potential of SH2 domain-containing inositol-5ʹ-phosphatase 1 (SHIP1) and SHIP2 inhibition in cancer. Mol Med.

[59] Matusik RJ, Jin RJ, Sun Q, Wang Y, Yu X, Gupta A, Nandana S, Case TC, Paul M, Mirosevich J, Oottamasathien S, Thomas J (2008). Prostate epithelial cell fate. Differentiation.

[60] Podlasek CA, Duboule D, Bushman W (1997). Male accessory sex organ morphogenesis is altered by loss of function of Hoxd-13. Dev Dyn.

[61] Mirosevich J, Gao N, Matusik RJ (2005). Expression of Foxa transcription factors in the developing and adult murine prostate. Prostate.

[62] Yu X, Wang Y, DeGraff DJ, Wills ML, Matusik RJ (2011). Wnt/β-catenin activation promotes prostate tumor progression in a mouse model. Oncogene.

[63] Schneider AJ, Moore RW, Branam AM, Abler LL, Keil KP, Mehta V, Vezina CM, Peterson RE (2014). In utero exposure to TCDD alters Wnt signaling during mouse prostate development: linking ventral prostate agenesis to downregulated β-catenin signaling. Toxicol Sci.

[64] Shahi P, Seethammagari MR, Valdez JM, Xin L, Spencer DM (2011). Wnt and Notch pathways have interrelated opposing roles on prostate progenitor cell proliferation and differentiation. Stem Cells.

[65] Wang BE, Wang XD, Ernst JA, Polakis P, Gao WQ (2015). Regulation of epithelial branching morphogenesis and cancer cell growth of the prostate by Wnt signaling. PLoS One.

[66] Jonsson M, Björntorp Mark E, Brantsing C, Brandner JM, Lindahl A, Asp J (2004). Hash4, a novel human achaete-scute homologue found in fetal skin. Genomics.

[67] Raposo AA, Vasconcelos FF, Drechsel D, Marie C, Johnston C, Dolle D, Bithell A, Gillotin S, van den Berg DL, Ettwiller L, Flicek P, Crawford GE, Parras CM (2015). Ascl1 coordinately regulates gene expression and the chromatin landscape during neurogenesis. Cell Rep.

[68] Rheinbay E, Suvà ML, Gillespie SM, Wakimoto H, Patel AP, Shahid M, Oksuz O, Rabkin SD, Martuza RL, Rivera MN, Louis DN, Kasif S, Chi AS (2013). An aberrant transcription factor network essential for Wnt signaling and stem cell maintenance in glioblastoma. Cell Rep.

[69] Hurley PJ, Marchionni L, Simons BW, Ross AE, Peskoe SB, Miller RM, Erho N, Vergara IA, Ghadessi M, Huang Z, Gurel B, Park BH, Davicioni E (2012). Secreted protein, acidic and rich in cysteine-like 1 (SPARCL1) is down regulated in aggressive prostate cancers and is prognostic for poor clinical outcome. Proc Natl Acad Sci USA.

[70] Hurley PJ, Hughes RM, Simons BW, Huang J, Miller RM, Shinder B, Haffner MC, Esopi D, Kimura Y, Jabbari J, Ross AE, Erho N, Vergara IA (2015). Androgen-regulated SPARCL1 in the tumor microenvironment inhibits metastatic progression. Cancer Res.

[71] Zheng L, Sun D, Fan W, Zhang Z, Li Q, Jiang T (2015). Diagnostic value of SFRP1 as a favorable predictive and prognostic biomarker in patients with prostate cancer. PLoS One.

[72] Dong W, Feng L, Xie Y, Zhang H, Wu Y (2011). Hypermethylation-mediated reduction of LMX1A expression in gastric cancer. Cancer Sci.

[73] Liu CY, Chao TK, Su PH, Lee HY, Shih YL, Su HY, Chu TY, Yu MH, Lin YW, Lai HC (2009). Characterization of LMX-1A as a metastasis suppressor in cervical cancer. J Pathol.

[74] Chao TK, Yo YT, Liao YP, Wang YC, Su PH, Huang TS, Lai HC (2013). LIM-homeobox transcription factor 1, alpha (LMX1A) inhibits tumourigenesis, epithelial-mesenchymal transition and stem-like properties of epithelial ovarian cancer. Gynecol Oncol.

[75] Luo Y, Kaz AM, Kanngurn S, Welsch P, Morris SM, Wang J, Lutterbaugh JD, Markowitz SD, Grady WM (2013). NTRK3 is a potential tumor suppressor gene commonly inactivated by epigenetic mechanisms in colorectal cancer. PLoS Genet.

[76] Genevois AL, Ichim G, Coissieux MM, Lambert MP, Lavial F, Goldschneider D, Jarrosson-Wuilleme L, Lepinasse F, Gouysse G, Herceg Z, Scoazec JY, Tauszig-Delamasure S (2013). Dependence receptor TrkC is a putative colon cancer tumor suppressor. Proc Natl Acad Sci USA.

[77] Schaeffer EM, Marchionni L, Huang Z, Simons B, Blackman A, Yu W, Parmigiani G, Berman DM (2008). Androgen-induced programs for prostate epithelial growth and invasion arise in embryogenesis and are reactivated in cancer. Oncogene.

[78] Spike BT, Engle DD, Lin JC, Cheung SK, La J, Wahl GM (2012). A mammary stem cell population identified and characterized in late embryogenesis reveals similarities to human breast cancer. Cell Stem Cell.

[79] Edgar R, Domrachev M, Lash AE (2002). Gene expression Omnibus: NCBI gene expression and hybridization array data repository. Nucleic Acids Res.

