# Supplementary material for: s-SHIP expression identifies a subset of murine basal prostate cells as neonatal stem cells
Source: Oncotarget. 2016 Apr 12;7(20):29228–44. doi: 10.18632/oncotarget.8709 (PMC5045392; doi:10.18632/oncotarget.8709)
Supplement: Supplementary file 1 [file oncotarget-07-29228-s001.pdf]

s-SHIP expression identifies a subset of murine basal prostate cells as neonatal stem cells

Supplementary Materials

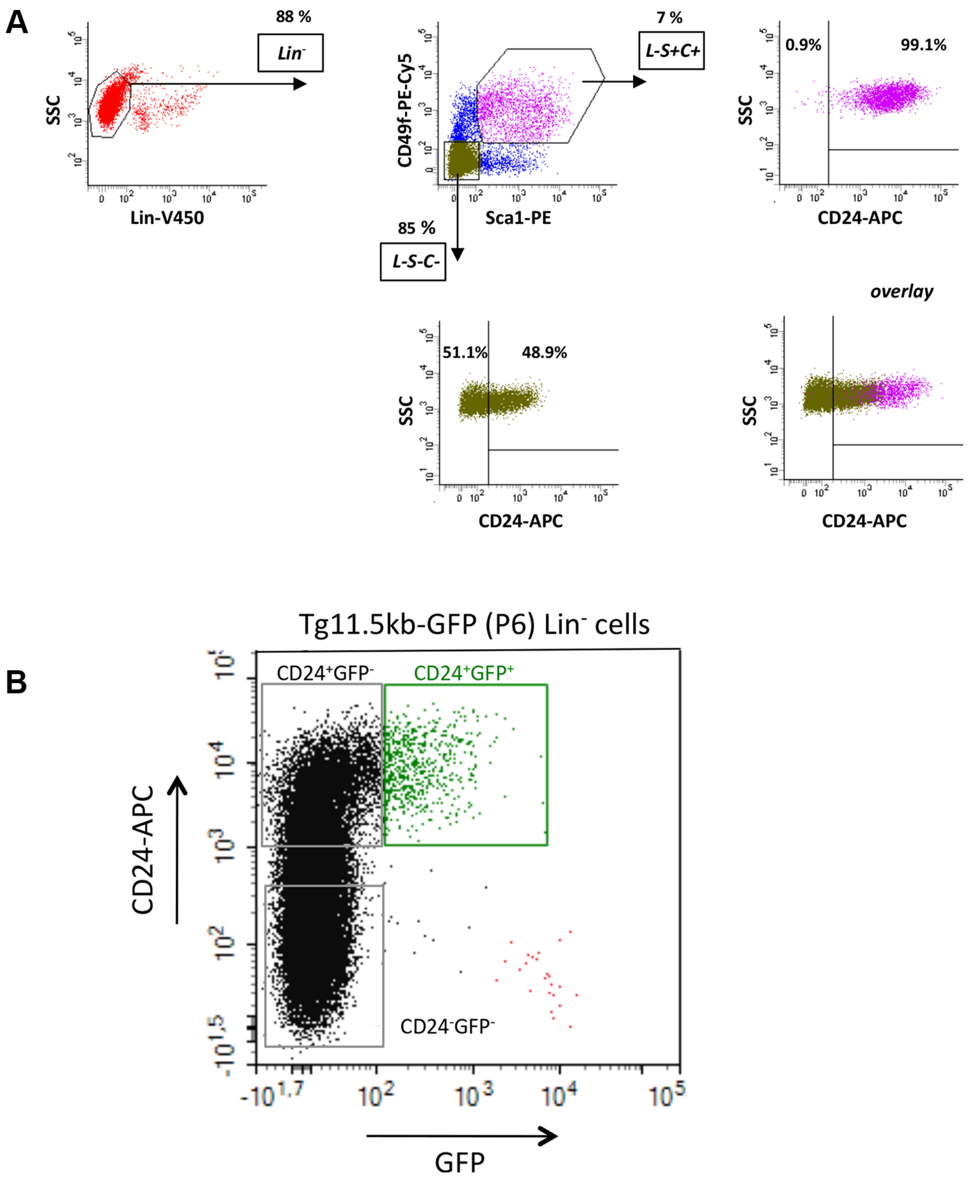

Supplementary Figure S1: (**A**) L-S+C+ prostate cells from P6 Tg 11.5kb-GFP mice express the CD24$^+$ prostate epithelial marker. Representative flow cytometry analysis (n = 3) of dissociated prostate cells stained for cell lineage markers, Sca-1, CD49f, and CD24; FACS plots show CD24 expression of gated L-S+C+ cells (upper-right panel) and gated L-S-C- cells (lower-middle panel). (**B**) FACS plot shows gates drawn for cell sorting of Lin$^-$ CD24$^-$ GFP$^-$, Lin$^-$ CD24$^+$ GFP$^-$ and Lin$^-$ CD24$^+$ GFP$^+$ cell subpopulations. Representative flow cytometry analysis (n > 5) of dissociated prostate cells isolated from P6 Tg 11.5kb-GFP mice and stained for cell lineage markers, and CD24. Side-scatter (SSC), phycoerythrin (PE), phycoerythrin-cyanine 5 (PE- Cy5), allophycocyanin (APC).

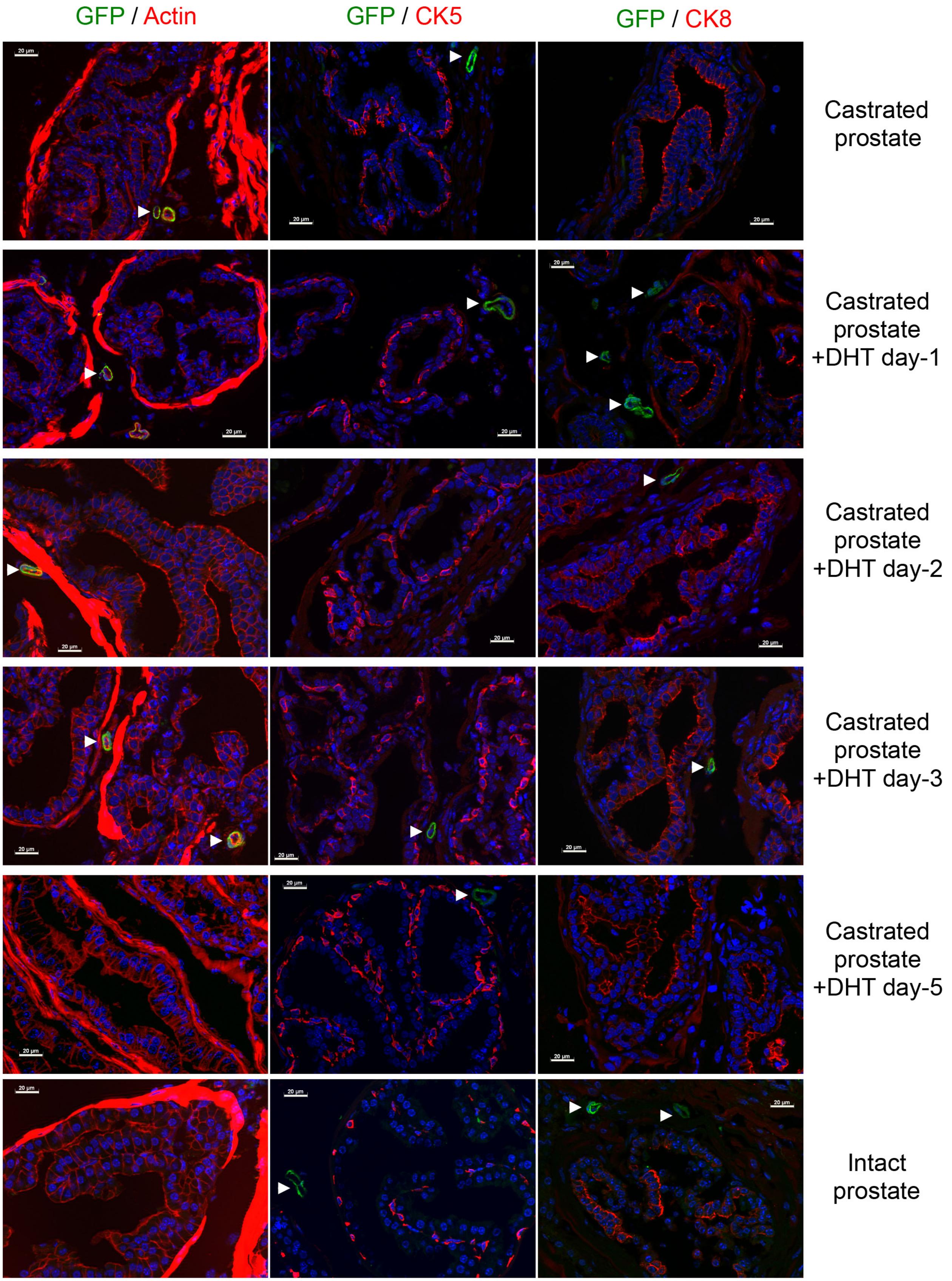

Supplementary Figure S2: In the regenerated prostate, only GFP+ vascular smooth muscle cells are detectable. Representative photographs (*n* = 3) of immunofluorescent staining (red) of prostate tissue frozen sections for actin (left panels), cytokeratin 5 (CK5) basal cell marker (middle panels), and cytokeratin 8 (CK8) luminal cell marker (right panels) ; GFP+ cells were only observed around blood vessels (arrowhead). Sections were counterstained with DAPI nuclear stain (blue). Scale bar: 20 µm.

A

Prostate cell lines

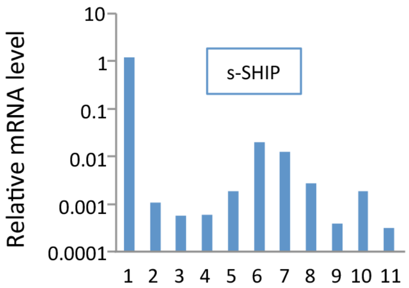

sample	cell line
1	RWPE-1
2	CWR-22RVI
3	DU145
4	LAPC4
5	LNCaP
6	P69
7	M12
8	MDA-Pca-2b
9	PC3
10	NCI-H660
11	V-CaP

LuCaP xenografts

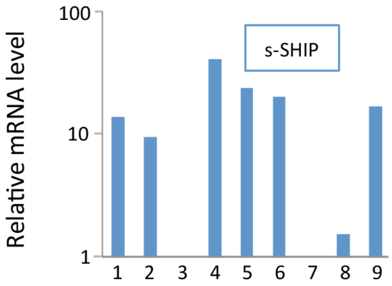

sample	LuCaP #	Tissue Type	Androgen status
1	23,1	Lymph node	dependant
2	23.1AI	Lymph node	independent
3	35	Inguinal Lymph Node	dependant
4	49	Omental fat metastasis	independent
5	78	Peritoneal metastasis	ND
6	81	R. Pelvic Lymph Node	independent
7	86.2	Bladder metastasis	independent
8	96	TURP	dependant
9	136	Acites fluid (cells)	dependant

B

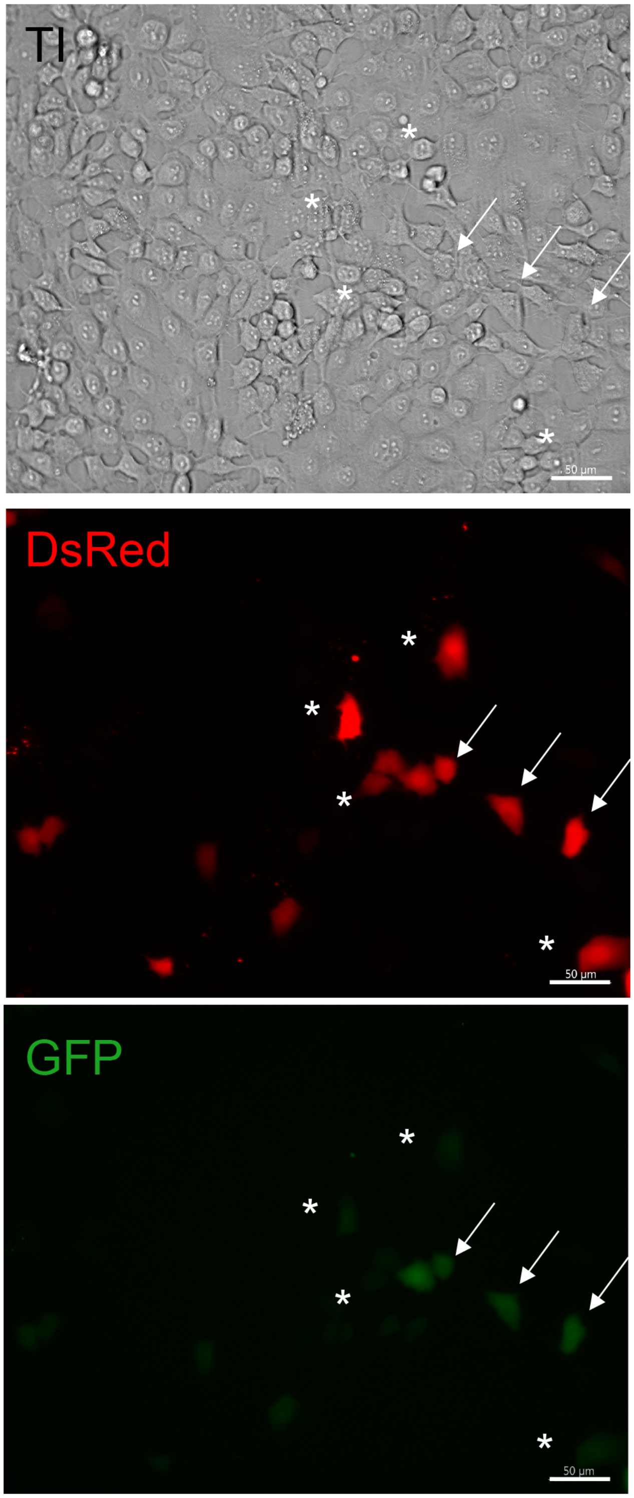

Supplementary Figure S3: (**A**) Human s-SHIP mRNA is expressed in various prostate cancer cell lines and xenograft tumors. Quantitative RT-PCR analysis of s-SHIP expression using total RNA isolated from 11 prostate cancer cell lines (left) and 9 xenograft tumors (right). The SYBR green method was used and the experimental Ct was calibrated against that of the RPLPO control product. For prostate cell lines, the expression values were ajusted by setting the s-SHIP relative expression of RWPE-1 cells to be 1. Data are representative of two independent experiments. LuCaP xenografts are from different tumors that have been propagated in immunocompromised mice. 23.1AI is the androgen insensitive variant of 23.1 that survived after the mouse has been castrated. (**B**) Heterogeneity of s-SHIP promoter expression of prostate cancer cells. M12 prostate cancer cells were transfected with a plasmid containing both s-SHIP-GFP promoter reporter and CMV promoter-DsRed. CMV-DsRed was obtained as a PCR fragment of pDsRed-monomer-N1 (Clontech), and inserted at the Not1 site of pBSK-11.5-kb-GFP plasmid [26]. Heterogeneity of GFP/s-SHIP expression was observed in the transfected DsRed- expressing cell population, with DsRed^{+}GFP^{-} (asterisks) and DsRed^{+}GFP^{+} (arrows) cells. Scale bars : 50 µm.

Supplementary Material List

Liste 1 : Primers used for RT-qPCR
LEF1 : forward : 5′-CGAGACAATTATGGCAAGAAGAAG-3′ ; reverse : 5′- GCTTCTCTTACCACCATGTTTCAG-3′
ROR1 : forward : 5′- CATACAAGCCCAAGAGCAAG-3′ ; reverse : 5′- GGCCCTTATAGATTTTTCCAAAG-3′
WNT10B : forward : 5′-GCAAGCTGGTGGTAACGG-3′ ; reverse : 5′- CTCAGTGCTGCCCCGATG-3′
